# Functional genomic analysis of the 68-1 RhCMV-*Mycobacteria tuberculosis* vaccine reveals an IL-15 response signature that is conserved with vector attenuation

**DOI:** 10.3389/fimmu.2024.1460344

**Published:** 2024-10-15

**Authors:** Cheng-Jung Sung, Leanne S. Whitmore, Elise Smith, Jean Chang, Jennifer Tisoncik-Go, Aaron Barber-Axthelm, Andrea Selseth, Shana Feltham, Sohita Ojha, Scott G. Hansen, Louis J. Picker, Michael Gale

**Affiliations:** ^1^ Center for Innate Immunity and Immune Disease, Department of Immunology, University of Washington, Seattle, WA, United States; ^2^ Washington National Primate Research Center, Seattle, WA, United States; ^3^ Vaccine and Gene Therapy Institute and Oregon National Primate Research Center, Oregon Health and Science University, Beaverton, OR, United States

**Keywords:** tuberculosis, cytomegalovirus, rhesus macaque, vaccine, spread-deficient, IL-15, innate immunity, transcriptomic analysis

## Abstract

Tuberculosis (TB), caused by *Mycobacterium tuberculosis* (*Mtb*) is a deadly infectious disease having a major impact on global health. Using the CMV vector for development of novel vaccines is a promising new strategy that elicits strong and durable, high frequency memory T cell responses against heterologous immunogens. We conducted functional transcriptomic analysis of whole blood samples collected from cohorts of rhesus (Rh) macaques that were administered RhCMV/TB vector using a prime-boost strategy. Two modified CMV vectors were used in this study, including 68-1 RhCMV/TB-6Ag (encoding 6 *Mtb* protein immunogens, including Ag85A, ESAT-6, Rv3407, Rv2626, Rpf A, and Rpf D) and its attenuated variant, 68-1 RhCMV/Δpp71-TB-6Ag (a cell-to-cell spread-deficient vaccine vector lacking the Rh110 gene encoding the pp71 tegument protein). Bulk mRNA sequencing, differential gene expression, and functional enrichment analyses showed that these RhCMV/TB vaccines induce the innate and adaptive immune responses with specific transcriptomic signatures, including the IL-15-induced protective gene signature previously defined to be linked with protection against simian immunodeficiency virus (SIV) by the 68-1 RhCMV/SIV vaccine. While both vectors exhibited a transcriptomic response of the IL-15 protective signature in whole blood, we show that lack of pp71 does not maintain induction of the protective signature for the full duration of the study compared to the parental non-attenuated vector. Our observations indicate that RhCMV vector vaccines induce a transcriptomic response in whole blood that include a conserved IL-15 signature of which vector-encoded pp71 is an important component of response durability that upon future *Mtb* challenge may define specific vaccine protection outcomes against *Mtb* infection.

## 1 Introduction

*Mycobacterium tuberculosis* (*Mtb*) primarily infects the lungs causing tuberculosis (TB) with an estimated 1.3 million deaths in 2022 and a death rate of around 50% if untreated (1). For individuals who are human immunodeficiency virus (HIV)-positive, TB is particularly devastating as it is the leading cause of death for people infected with both pathogens. With the increasing prevalence of drug-resistant TB strains, finding a protective vaccine solution is essential for reducing the global infection and disease rates (2). Currently, there is only one licensed vaccine in use for TB control, Bacillus Calmette-Guérin (BCG) vaccine, underscoring the need for investigation of novel vaccines to prevent pulmonary TB disease.

HIV causes disease by infecting and depleting CD4^+^ T cells. CD8^+^ T cells play a crucial role to control HIV by facilitating cytolytic mechanism to remove infected CD4^+^ T cells. The cytomegalovirus (CMV) vector with its unique ability to elicit a strong and durable CD8^+^ T cell response has shown great promise for use as a novel vaccine vector, particularly for HIV. Simian immunodeficiency virus (SIV), a lentivirus that infects nonhuman primates such as rhesus macaques (RMs), exhibits infection and pathogenic response similar to HIV with CD4^+^ T cell depletion. The 68-1 derived RhCMV/SIV vector has the unique ability to control and clear SIV (3–5) in RMs by quickly eliciting and maintaining a high level of effector T cells with SIV-specific recognition by T cells occurring through MHC-Ia, MHC-II, or MHC-E antigens. Specifically, MHC-E restricted CD8^+^ cells facilitate clearance of SIV (5). This SIV control and clearance behavior was also observed in the attenuated version of the 68-1 RhCMV/SIV vaccine (68-1 RhCMV/SIV Δpp71) that lacks expression of viral protein that facilitates CMV spread and dissemination through the body, thereby enhancing vector safety (6). CD8^+^ T cells induction and regulation can be triggered by multiple factors, including specific antigens and cytokines such as IL-12, IL-18, and IL-15. Many studies have shown that IL-15 promotes memory CD8^+^ T cells homeostatic proliferation to expand CD8^+^ T cells, resulting in enhanced immunity against diseases including SIV and TB. Functional genomic analysis via mRNA-sequencing (Seq) of whole blood in 68-1 RhCMV/SIV-vaccinated RMs identified an IL-15-mediated transcriptional response that linked with vaccine protection against multiple SIV challenges (4).

This RhCMV vaccine backbone has also shown promising results in the prevention of *Mtb* infection and disease. Previous *Mtb* challenge studies have demonstrated that RhCMV/TB vaccination resulted in circulating *Mtb*-specific CD4^+^ and CD8^+^ T cells, and RMs with subcutaneous RhCMV/TB vaccination showed reduced *Mtb* infection and disease (based on both *Mtb* culture and pathologic score) by nearly 70% compared to unvaccinated controls (7). In these studies, functional transcriptomic analysis of whole blood post *Mtb* challenge revealed vaccine-induced innate immune responses linked with reduced disease (7). Here, we administered a prime plus two boosts regimen of the 68-1 RhCMV/TB-6Ag and 68-1 RhCMV/Δpp71-TB-6Ag vaccines to independent cohorts of 12 and eight RMs, respectively. These vaccines with RhCMV vectors encoding a single six-antigen-expressing polyprotein insert consist of two antigens from each of these three classes: acute-phase (ESAT-6 and Ag85A), latency (Rv3407 and Rv2626), and resuscitation (Rpf A and Rpf D; GenBank KY611405). These six antigens were selected by Hansen et al. in 2018 (7) using a mouse challenge model in which each antigen was shown to be at least partially protective. We conducted mRNA-sequencing of whole blood to define gene expression correlates of the response elicited by each vaccine. Differential gene expression and functional enrichment analyses of the transcriptomic signature of whole blood show that both vectors induce specific innate and adaptive immune response signatures of myeloid cell function, T cell receptor signaling, and lymphocyte activation. Unique signatures are linked with each vaccine. We show that the RhCMV/Δpp71-TB-6Ag vaccine transcriptional response wanes after the second boost. Direct comparison of RhCMV/TB vaccine signatures with the RhCMV/SIV vaccine signature (4) revealed similar transcriptional responses featuring IL-15-linked protective signature response. Our study shows that IL-15 signature is a major component of immune programming by 68-1 RhCMV vectored vaccines.

## 2 Results

### 2.1 Initial examination of sample variation across vaccine cohorts

**Figure 1:**
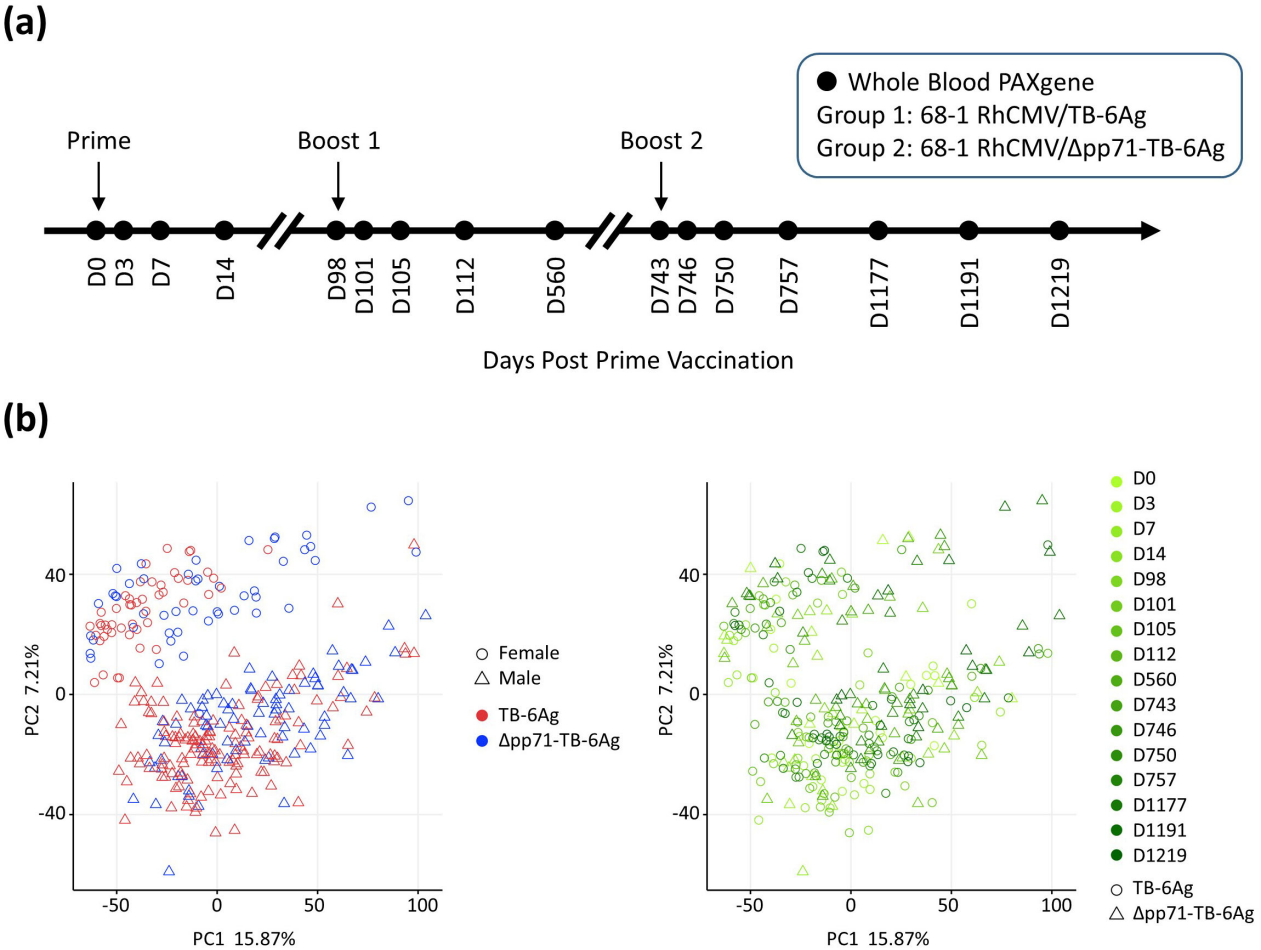
NHP vaccine study design and PCA describing the general variance. **(A)** Schematic overview of the two vaccine groups, vaccination time, and the 16 time points for which whole blood samples were collected for RNA-seq analysis in this study. Arrows indicated time points when RMs were administered with either the 68-1 RhCMV/TB-6Ag or the 68-1 RhCMV/Δpp71-TB-6Ag vectors set at D0 (prime), D98 (boost 1), and D743 (boost 2). **(B)** PCA biplots showing transcriptomic variation across all samples in this study. Each point on the plot represented an RNA-seq sample from an animal at each time point during vaccination regimen, for the visualization of vaccine group versus sex (left) and vaccine group versus time point (right). In the left panel, shape denoted sex (circle for female; triangle for male) and color denoted vaccine type (red for 68-1 RhCMV/TB-6Ag; blue for 68-1 RhCMV/Δpp71-TB-6Ag). In the right panel, color denoted time point (light to dark green for D0 to D1219) and shape denoted vaccine type (circle for 68-1 RhCMV/TB-6Ag; triangle for 68-1 RhCMV/Δpp71-TB-6Ag).

To examine the differences in the vaccine signatures between RhCMV/TB vaccines, we conducted a functional genomic analysis of whole blood collected from immunized RM (*Macaca mulatta*) using bulk mRNA sequencing (mRNA-Seq). A total of 24 RMs (males and females) were randomized and assigned to one of two vaccine groups (n=12 per group). Animals received a prime-boost vaccine regimen of 68-1 RhCMV/TB-6Ag (n=12) or 68-1 RhCMV/Δpp71-TB-6Ag vector (n=12) spaced 14 weeks apart that was followed by a second boost administered 92 weeks later (**Figure 1A**, **Supplementary Table S1**). Four RMs of 68-1 RhCMV/Δpp71-TB-6Ag group were not included for the further transcriptional analyses due to lack of second boost data. Herein, we refer to these RhCMV vaccine vectors as non-attenuated (68-1 RhCMV/TB-6Ag) and attenuated (68-1 RhCMV/Δpp71-TB-6Ag) vectors, respectively. Whole blood samples collected at pre-vaccination day 0 (D0) and 15 additional time points following prime and boost immunization during the vaccination phase of the study were processed for bulk mRNA-Seq analyses.

To initially assess the major sources of variation within the resulting mRNA-Seq dataset, principal component analysis (PCA) was performed using all 15,399 expressed genes for 320 samples (20 RMs, each with 16 time points). A major source of variation was the sex of the animals, along principal component (PC)2 (**Figure 1B**). We accounted for this variation in downstream differential gene expression analysis by making sex a covariate in our linear model, thus removing batch effects due to sex in order to increase overall power of our study. Analysis of sex-specific vaccine response differences will be directly addressed in a subsequent study. PCA also revealed that samples neither segregated by time point nor by vaccine type (**Figure 1B**), reflecting a conserved response across vaccines.

### 2.2 Complete blood count and deconvolution analysis

**Figure 2:**
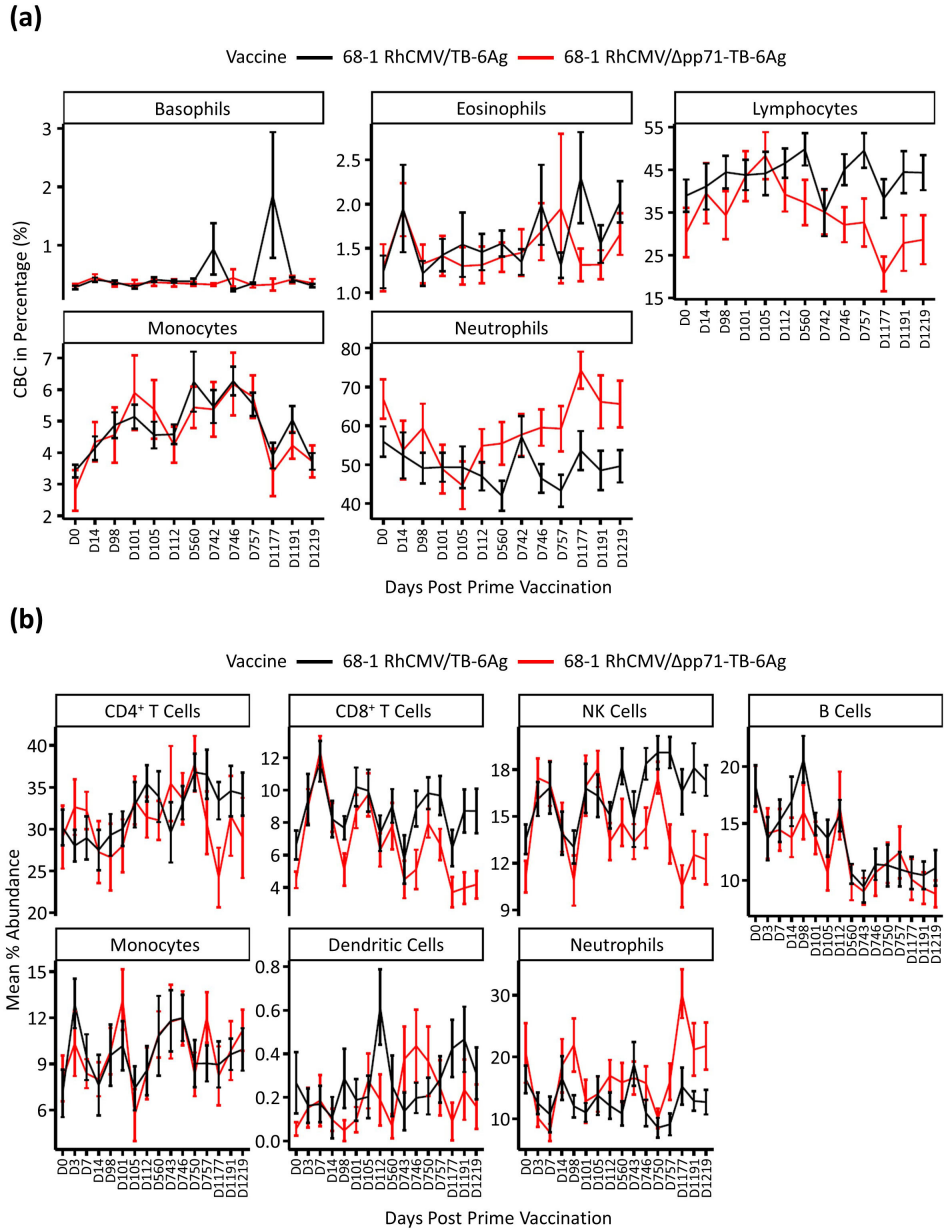
Cell type abundance analysis. **(A)** Complete blood count (CBC) test in percentage of five cell types, including basophil, eosinophil, lymphocyte, monocyte, and neutrophil. Vaccine groups were labeled in two colors, where black represented 68-1 RhCMV/TB-6Ag and red represented 68-1 RhCMV/Δpp71-TB-6Ag. Plots were made using mean data with standard error bars for each group at 13 time points post prime vaccination. **(B)** Deconvolution analysis of RNA sequencing data by CIBERSORTx to predict cell type abundance. Vaccine groups were labeled in the same way as described above in **(A)**, and plots were made using mean data with standard error bars for each group at all 16 time points post prime vaccination.

Initially we examined complete blood count (CBC) data collected from across the vaccine time course for an overview of blood cell count dynamics of the different vaccine vector groups (**Figure 2A**). Generally, lymphocytes and neutrophils showed a separation of cell proportions between the two vaccine vector groups after the second boost vaccination at day (D)743. Specifically, compared to baseline, lymphocytes (including T, B, and NK cells) showed a decreasing trend in abundance in the attenuated vaccine vector group while staying stable in the non-attenuated vaccine vector group, with increased neutrophils in the attenuated vaccine group. We also performed a deconvolution analysis of the whole blood transcriptional dataset using CIBERSORTx with the LM22 signature (8, 9) to predict the abundance of different cell types in each sample and to interrogate differences observed across time points and vaccine vector groups. Cell abundance averages and standard error were calculated for each time point within the two groups (**Figure 2B**). We observed an initial increase in CD8^+^ T and NK cells following prime and the first boost administrations in both vaccine groups. Following the second boost, NK and CD8^+^ T cells in the attenuated group decreased to D0 abundance levels while remaining elevated in animals who received the non-attenuated vaccine vector. These observations show that the non-attenuated vaccine vector caused more persistent and sustained change in NK and CD8^+^ T cell abundances compared to the attenuated vaccine vector.

### 2.3 Global analysis of whole blood transcriptomic signature induced by RhCMV/TB vaccination across vector types

**Figure 3:**
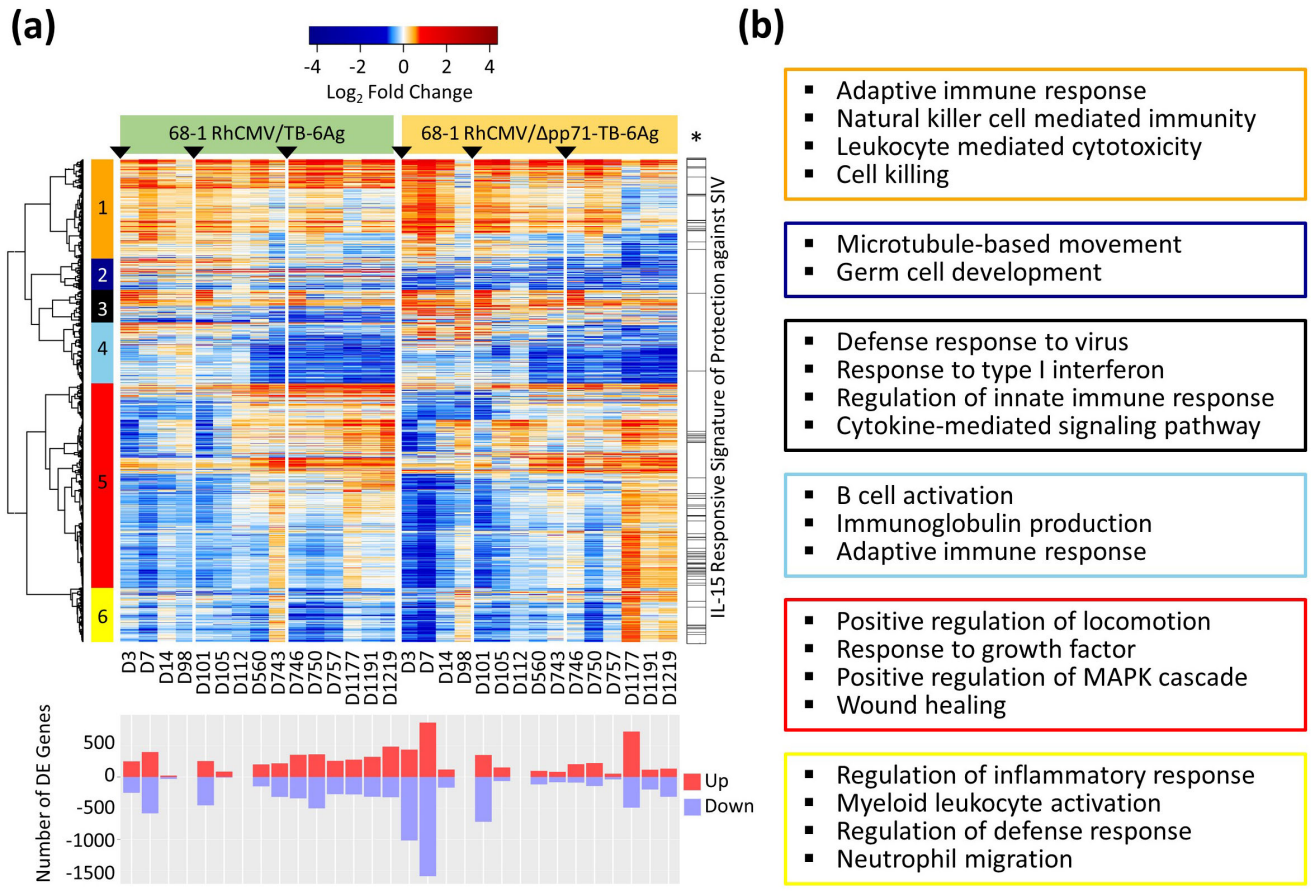
Whole blood mRNA-Seq analysis of RhCMV/TB vaccine response in RMs. **(A)** Heatmap of LFC values of all 4,816 significant (FDR-adjusted *p*-value < 0.05 and |log_2_FC| > 0.58) DE genes identified in at least one vaccine group and one time point for both the non-attenuated (left side) and the attenuated (right side) vaccine groups with a gradient color intensity bar above the heatmap indicating the LFC values where red and blue depicted as up- and down-regulated, respectively. Barplot below the heatmap showed the total number of significant DE genes at each time point relative to baseline (D0), with up- and down-regulated genes labeled separately. Six gene modules were defined using Pearson correlation followed by Ward.D2 hierarchical clustering method. The bar on the right of the heatmap with an asterisk label (*) showed the genes overlapping the previously published protection signature. **(B)** Functional enrichment analyses on DE gene modules were performed for each cluster in heatmap. Select significant biological processes for each cluster were listed in colored boxes matching to the clusters in the heatmap (orange, darkblue, black, skyblue, red, and yellow, for clusters 1 to 6, respectively).

To identify the whole blood gene expression responses to the RhCMV/TB vaccines, we performed differentially expressed (DE) analyses to define genes with statistically significant expression changes from D0 at each time point in both vaccine vector groups. A total of 4,816 significant DE genes were defined in at least one time point post-vaccination, with a false discovery rate (FDR; Benjamini-Hochberg (BH) procedure)-adjusted *p*-value less than 0.05 and an absolute log_2_ fold change (LFC) value greater than 0.58 (equivalent to fold change 1.5). Transcriptional changes strongly increased after each immunization (prime, boost 1, and boost 2) which then decreased over time (**Figure 3A**, **Supplementary Table S2**). This outcome shows that the vaccine responses induced by the RhCMV/TB vaccine vectors are rapidly induced. All 4,816 significant DE genes were assessed by cluster analysis using Pearson correlation and Ward.D2 hierarchical clustering methods which revealed six distinct gene modules. Overall, the whole blood transcriptional kinetics of the response of both vaccine vector groups appeared similar, except that the non-attenuated vaccine vector induced a stronger response that was prolonged and maintained over 92 weeks through D1219 following prime immunization as compared to the response elicited by the attenuated vaccine vector group that was less durable and diminished at D1177 post-prime immunization. Compared to the non-attenuated vaccine vector group, many DE genes from clusters 1, 5, and 6 lost their expression signatures after approximately three years following the prime immunization (D1177, D1191, and D1219) in the attenuated vaccine vector group. These results demonstrated the durability differences of the vaccine vector responses, revealing that the durability of the 68-1 RhCMV/Δpp71-TB-6Ag-induced response wanes within 3-years post-vaccination. Thus, the RhCMV pp71 protein is important for long-term maintenance of the vaccine transcriptional response.

Previously, we identified a RhCMV/SIV vaccine whole blood protective transcriptomic signature of 186 genes that were part of the response to IL-15, expressed in vaccinated RMs who were protected against SIV infection (4). We evaluated if any of the genes in this protection signature were present in the DE genes of each RhCMV/TB vaccine vector examined in this study. Of the 186 protection signature genes featuring the response to IL-15, 131 (70.43%) were identified as significant DE genes across the vaccine vectors in this study (**Figure 3A**, right hand side, **Supplementary Table S3**), showing that the 68-1 RhCMV vaccine vector generates a conserved signature that includes an IL-15 response program in whole blood.

Other relevant biological processes associated with these DE genes were investigated using over-representation analysis (ORA) performed on each of the six modules of genes identified by clustering. Select significant biological processes are shown in **Figure 3B** (the full set of significant enrichments are shown in **Supplementary Table S4**). Genes that were induced after prime immunization with both RhCMV/TB vaccine vectors were mostly found in cluster 1, with specific gene expression then variably reduced at late time points (D1177, D1191, and D1219) especially for the attenuated vaccine vector group. We found enrichment for genes in cluster 1 related to adaptive immune response programming (e.g., *CX3CR1*, *GATA3*, *IFNG*, *IL10*, *IL18*, *IL23A*, *PRF1*, and *TBX21*), NK cell-mediated immunity, and leukocyte-mediated cytotoxicity. Cluster 5 included the greatest number of co-expressed genes. Notably, most of these genes were reduced in expression compared to baseline levels (down-regulated) after the prime vaccination with the expression increasing over time for both vaccine groups. For this cluster, the attenuated vaccine vector group showed a stronger magnitude of expression increase at D1177, D1191, and D1219 that linked to the reduced expression of the otherwise induced module 1 genes. Cluster 5 genes were enriched for fundamental cellular processes including regulation of MAPK signaling and featuring *CCR1*, *MAP3K5*, *MYD88*, *PIK3CG*, and *TLR4*. Cluster 6 genes were typically down-regulated from baseline following vaccination but for animals who received the attenuated vaccine vector the expression of these genes shifted at the later time points to levels of induction/up-regulation compared to baseline. Module 6 genes were significantly associated with defense related innate immune response and regulation (e.g., *CASP4*, *IL1RL1*, *MAPK13*, *STAT5B*, and *TRIM5*), including myeloid leukocyte activation and neutrophil migration. Many of the DE genes in clusters 1, 5, and 6 showed differences between the attenuated and non-attenuated vaccine groups three years following the prime immunization (D1177, D1191, and D1219) and linked with the protection signature defined by Barrenäs et al. in 2021 (4; see **Figure 3A**, right), showing that the genes in the protection signature were similarly regulated during the prime and boost 1 phases for both vaccine vectors but this response became dysregulated during the boost 2 phase in animals who received the attenuated vaccine vector.

We also found that genes in cluster 2 (see **Figure 3A**) were associated with cilium movement and microtubule-based movement defining regulation of cell motility, and cell trafficking (e.g., *CFAP45*, *DNAH1*, *KIF14*, *RNASE10*, *TSSK4*, and *TTC21A*). Cluster 2 genes were variably expressed in animals who received the non-attenuated vaccine vector but were down-regulated in the attenuated vaccine vector animals across time points. Genes in cluster 3, including *CXCL9*, *CXCL10*, *IRF7*, *IRF9*, *STAT1*, *STAT2*, and *TRIM21*, were enriched for antiviral innate immune response related biological processes, such as response to type I interferon (IFNs) and cytokine-mediated signaling pathway, and these genes were rapidly induced in response to administration of both RhCMV/TB vaccine vectors. Enrichment for cluster 4, with significant genes such as *CD19*, *CD40*, *CXCR5*, *IGHE*, and *TLR9*, included humoral immune responses and were strongly down-regulated late in the vaccine phase for both groups.

### 2.4 Upstream regulator analysis and network of target genes

**Figure 4:**
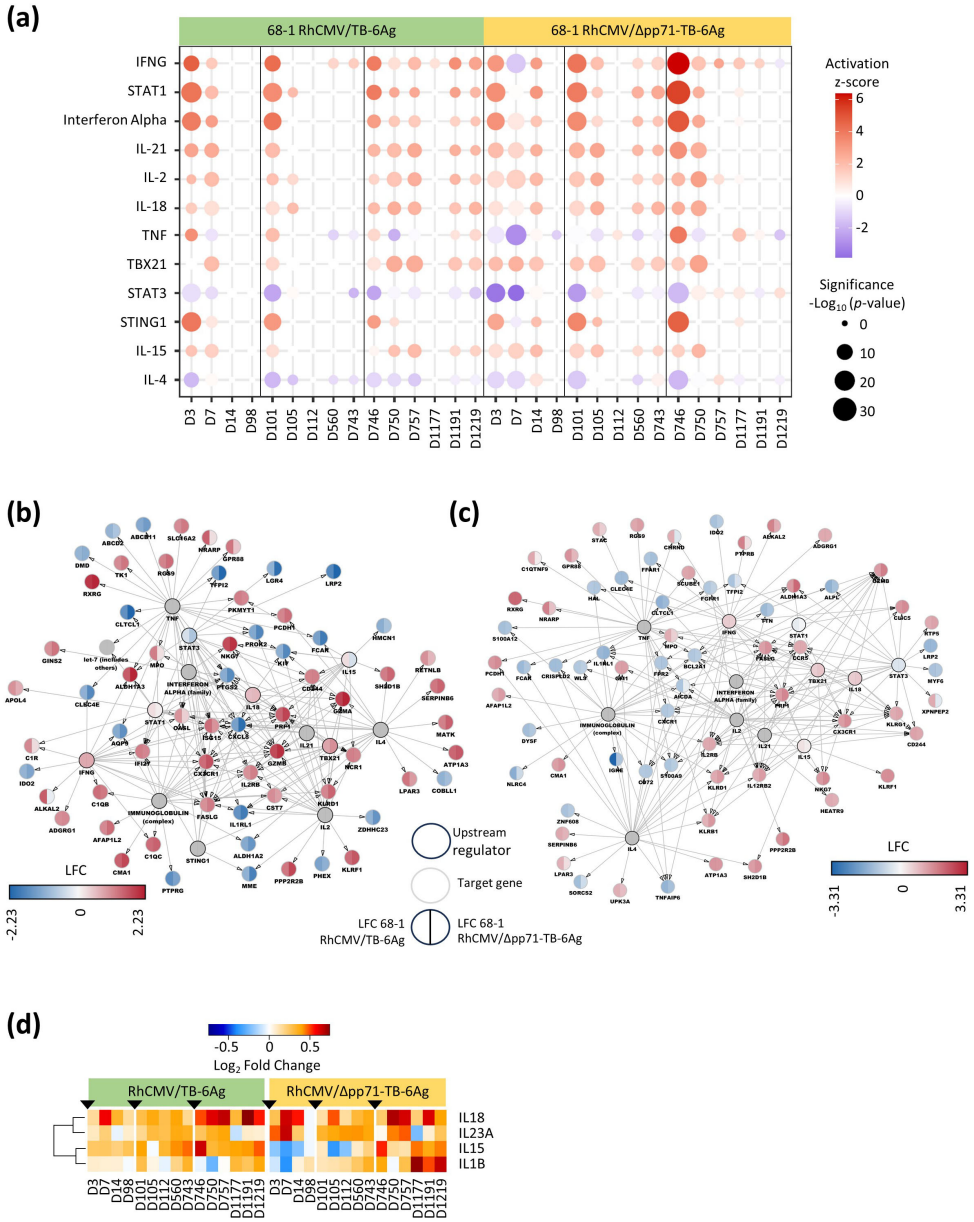
Upstream analysis and regulation networks in RhCMV/TB-vaccinated RMs. **(A)** Dot plot delineated select top upstream regulators identified through Ingenuity Pathway Analysis (IPA). The colors represented z-scores (> 2 for activation in red and < -2 for inhibition in blue) and dot size was proportional to the significance of log_10_ *p*-value as per the legend. **(B, C)** Networks showing LFC levels of downstream targeted genes and the major upstream regulators (circles with black lines) in 68-1 RhCMV/TB-6Ag (left half of each circle) and 68-1 RhCMV/Δpp71-TB-6Ag (right half of each circle) vaccine groups at D7 **(B)** and D750 **(C)**. **(D)** Heatmap showing the significant (FDR-adjusted *p*-value < 0.05 and |log_2_FC| > 0.58) gene expression changes from baseline of *IL15* and the other important interleukin upstream regulator genes (*IL18*, *IL23A*, and *IL1B*) for both the non-attenuated (left side) and the attenuated (right side) vaccine groups, with a gradient color intensity bar above the heatmap indicating the LFC values where red and blue depicted as up- and down-regulated, respectively.

We performed upstream regulator analyses on the DE genes across the vaccine vector time series using Ingenuity Pathway Analysis (IPA). In particular, we included a focus to assess the upstream regulators linked with programming each vaccine vector whole blood gene expression signature, including those linked with signature durability. Upstream regulators were identified and sorted by both the sum of absolute z-scores compared to baseline and the sum of -log_10_ (*p*-value) across time points in each vaccine vector group. The top 12 significant upstream regulators identified from both vaccine vector groups are shown in **Figure 4A** and **Supplementary Table S5**. Among these upstream regulators, IFNG (gamma), STAT1, interferon alpha, IL-21, IL-2, IL-18, TBX21, STING, and IL-15 were predicted to be actively signaling at multiple points in the time course. STAT3 and IL-4 were predicted to be inhibited compared to baseline. TNF showed inconsistent activation patterns across the two vaccine groups. During the prime and boost 1 phases, upstream regulators of the attenuated/68-1 RhCMV/Δpp71-TB-6Ag group maintained significant activation or inhibition throughout both phases while the non-attenuated/68-1 RhCMV/TB-6Ag group had a relative decrease in activation/inhibition of upstream regulators after D7 and D105, for the prime and boost 1 phases respectively. Conversely, the attenuated/68-1 RhCMV/Δpp71-TB-6Ag group during boost 2 (D746-D1219) exhibited little to no significant regulation of these upstream regulators at late time points following the boost 2 phase while the regulation of the same upstream regulators in the non-attenuated/68-1 RhCMV/TB-6Ag group was maintained. The distinct dynamics of upstream regulators including loss of activation in the attenuated/68-1 RhCMV/Δpp71-TB-6Ag group therefore underscores the vaccine vector signature durability differences of DE gene expression particularly during the second boost phase.

To reveal the gene networks and their expression dynamics linked with these upstream regulators, we made network plots showing upstream regulators (*p*-value less than 0.05) and their DE target genes at D7 (**Figure 4B**) and D750 with LFCs for both vaccine groups (**Figure 4C**). The networks displayed the most DE genes at D7, revealing consistent up- and down-regulation of target genes across both vaccine groups. Unique to D7, genes *AQP9*, *CST7*, *CXCL8*, *IFI27*, *ISG15*, *KIT*, *NCR1*, *OASL*, *PROK2*, and *PTG2* were all induced/up-regulated compared to baseline reflecting an innate immune and adaptive immune activation state that links with the response to *Mtb* antigens (10). At D750, genes *AICDA*, *BCL2A1*, *CCR5*, *CD72*, *CLIC5*, *CRISPLD2*, *CXCR1*, *FPR2*, *GFI1*, *IL12RB2*, *KLRB1*, *KLRG1*, *S100A9*, *TNFAIP6*, and *WLS* were uniquely all targets of upstream regulators and mark a TH1-polarized adaptive immune response (11, 12).

For each vaccine vector and in both networks, IL-15 was identified as an active upstream regulator. Expression and signaling of IL-15 was shown to be linked to RhCMV/SIV vaccine protection in previous study (4). We identified IL-15 and four additional cytokines that were significantly DE in our vaccine vector groups, which included *IL15*, *IL18*, *IL23A*, and *IL1B* (**Figure 4D**, **Supplementary Table S6**). IL-15 was induced, and expression was typically maintained across the time course in the non-attenuated/68-1 RhCMV/TB-6Ag group but its expression was low and variable across prime and boost-1 time series in the attenuated/68-1 RhCMV/Δpp71-TB-6Ag group. We also found that IL-18 expression was consistently induced across the time series for both vaccine vector groups, and that IL-1β was induced to high levels at the end of the time series following boost-2 in the attenuated/68-1 RhCMV/Δpp71-TB-6Ag group.

### 2.5 Conservation of IL-15 signature between 68-1 RhCMV vector vaccines

**Figure 5:**
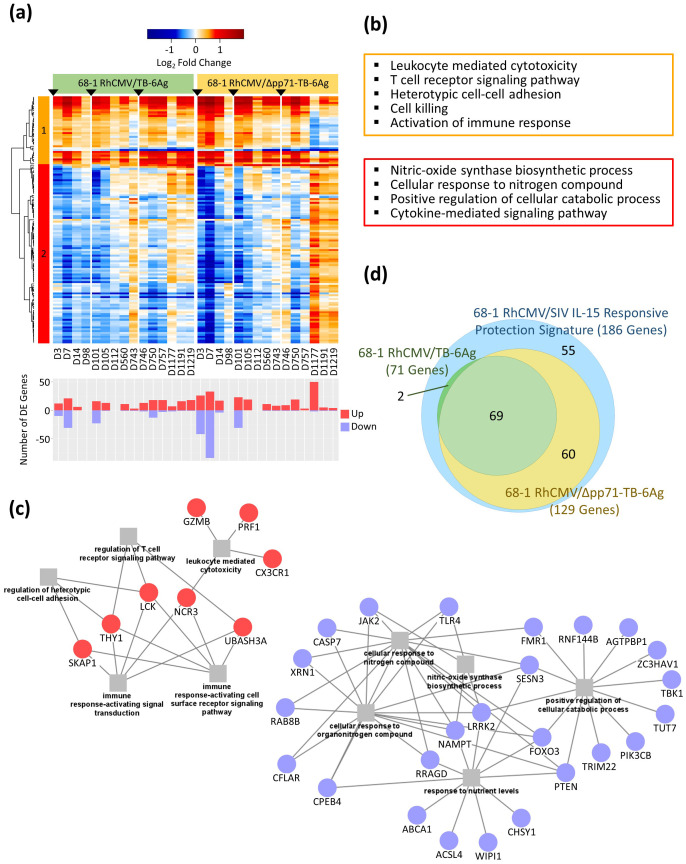
Conserved transcriptional profiles overlapping the IL-15 response signature of SIV protection. **(A)** Heatmap of the LFC values of 131 significant (FDR-adjusted *p*-value < 0.05 and |log_2_FC| > 0.58) DE genes overlapping the previously published IL-15 protection signature genes for both the non-attenuated (left side) and the attenuated (right side) vaccine groups, with a gradient color intensity bar above the heatmap indicating the LFC values where red and blue depicted as up- and down-regulated, respectively. Barplot below the heatmap showed the total number of significant DE genes at each time point relative to baseline (D0), with up- and down-regulated genes labeled separately. Genes were clustered into two modules using Pearson correlation followed by Ward.D2 hierarchical clustering method. **(B)** Functional enrichment analyses identified biological processes of genes significantly over-represented for each cluster according to colors of the clusters in the heatmap (orange for cluster 1; red for cluster 2). **(C)** Network showing select biological processes with significant overlapping genes for cluster 1 (left) and cluster 2 (right), with squares representing biological processes and circles showing the enriched DE genes. **(D)** Proportional Venn diagram of three circles, showing the overlap between the previously published IL-15 protection signature (186 genes in blue), overlapped 68-1 RhCMV/TB-6Ag vaccine signature (71 genes in green), and overlapped 68-1 RhCMV/Δpp71-TB-6Ag vaccine signature (129 genes in yellow).

We previously defined a 186 gene expression signature associated with RhCMV/SIV vaccine protection, all of which were shown to be regulated in response to IL-15 (4). Importantly, 131 of these genes were significantly DE across RhCMV/TB vaccine vectors (see **Figure 3A**). We evaluated the expression dynamics of this gene signature across the 68-1 RhCMV/SIV, non-attenuated/68-1 RhCMV/TB-6Ag and the attenuated/68-1 RhCMV/Δpp71-TB-6Ag vaccine vector responses (**Figure 5A**, **Supplementary Table S7**). Two gene modules were identified based on Pearson correlation and Ward.D2 hierarchical clustering including 36 genes in cluster 1 that were induced/up-regulated and 95 genes in cluster 2 that were typically suppressed/down-regulated compared to baseline. Genes in cluster 1 represented immune activation and immune effector functions and were immediately induced following prime vaccination. Expression of cluster 1 genes was typically maintained in the non-attenuated/68-1 RhCMV/TB-6Ag group but a subset of these genes demonstrated a transient down-regulation prior to boost 2, after which boost 2 restored their induced expression pattern. These genes underwent sustained reduction in expression in the attenuated/68-1 RhCMV/Δpp71-TB-6Ag group at late time points following boost 2. The down-regulated cluster 2 gene expression pattern shifted to increased levels of expression for a large subset of genes at late time points after each boost wherein this expression shift was more extensive and enhanced in the attenuated/68-1 RhCMV/Δpp71-TB-6Ag group. These results show that the non-attenuated/68-1 RhCMV/TB-6Ag and attenuated/68-1 RhCMV/Δpp71-TB-6Ag vaccine vectors each immediately induce the IL-15 response gene expression profile in blood. This signature is sustained and reengaged following boost but can wane in late time points following boost 2. Moreover, lack of vector-encoded pp71 impacts the longer-term durability and breadth of this gene expression signature.

We conducted enrichment tests using over representation analysis (ORA) methodology (13) to identify the biological processes associated with the 131 IL-15 response genes in our data sets. ORA of cluster 1 demonstrated an up-regulated enrichment of cell-mediated immune response related to immune signaling including leukocyte mediated cytotoxicity and the T cell receptor signaling pathway (**Figure 5B**, **Supplementary Table S8**), and produced gene networks with key genes such as *CX3CR1*, *GZMB*, *LCK*, *NCR3*, *PRF1*, *SKAP1*, *TBX21*, *THY1*, and *UBASH3A* (**Figure 5C**). The down-regulated genes in cluster 2 were significantly enriched for cellular response to oxygen levels, cytokine signaling, cell death, and catabolic processes, including genes such as *CASP7*, *FOXO3*, *JAK2*, *NAMPT*, *PIK3CB*, and *TRIM22* (**Figure 5C**; see **Supplementary Table S8** for the complete list of genes). Specifically, among these 131 RhCMV vector-induced signature genes, 71 were significantly DE in the non-attenuated/68-1 RhCMV/TB-6Ag group while 129 were significantly DE in the attenuated/68-1 RhCMV/Δpp71-TB-6Ag group, with 69 highly conserved genes identified to be significant in all three groups (protection signature, 68-1 RhCMV/TB-6Ag, and 68-1 RhCMV/Δpp71-TB-6Ag; **Figure 5D**, **Supplementary Table S3**). Importantly, these observations demonstrate that the IL-15 response protection signature identified by Barrenäs et al. in 2021 (4) was also induced in 68-1 RhCMV/TB vectors. Vaccine-induced regulation of these genes therefore links the functionality of RhCMV vaccines and could be important for vaccine efficacy.

## 3 Discussion

Here we present a functional genomic analysis of the RhCMV vaccine vectors including a non-attenuated/68-1 RhCMV/TB-6Ag vaccine vector and an attenuated/68-1 RhCMV/Δpp71-TB-6Ag vaccine vector with direct comparison to the RhCMV/SIV vaccine vector across prime/boost vaccination regimen. Both RhCMV/TB vectors express 6 specific *Mtb* antigens and were designed as vaccines to protect against infection and disease by *Mtb* (7), whereas the RhCMV/SIV vaccine expresses specific SIV antigens as a preclinical vaccine design for protection against SIV/HIV infection (14, 15). We found that similar to the RhCMV/SIV vaccine vector, the RhCMV/TB vaccine vectors (both 68-1 strain) rapidly induce the expression of genes that impart regulation of key immunological pathways programming cellular responses that impact both innate and adaptive immunity. Remarkably, the RhCMV vaccine vector whole blood signature is conserved across the three vectors analyzed here to feature a common IL-15 response signature that we previously linked with vaccine protection against SIV (4). While this vaccine vector signature exhibited fluctuating durability beyond three years for the non-attenuated/68-1 RhCMV/TB-6Ag vaccine vector, the vaccine signature was found to decrease following the second boost more strongly for the attenuated/68-1 RhCMV/Δpp71-TB-6Ag vaccine vector. These results indicate that the two *Mtb* vaccine vectors elicit similar responses of immune programming but with differential dynamics and overall durability linked with the RhCMV pp71 protein. Importantly, the non-attenuated/68-1 RhCMV/TB-6Ag vaccine vector can protect against infection and disease by *Mtb* (7).

We identified enriched upstream regulators from significantly DE genes in response to the 68-1 RhCMV/TB vaccines, including cytokines (e.g., IL-15, IL-2, IL-21, IL-18, and IL-4, IFN gamma) and transcription factors (e.g., STAT1, TNF, TBX21, STAT5, and STAT3). In focusing on target genes at D7 and D750, we identified innate immune genes (for example *IFI27* and *ISG15*) induced by more than three upstream regulators specifically at D7. This pattern of gene expression suggests that the vaccine elicited innate immune activation and responses through multiple pathways during the prime phase of the vaccination. Our data sets show that deletion of Rh110 encoding pp71 links with an inability of the attenuated vector to maintain the persistent activation of the upstream regulators and subsequent expression of specific target genes that support vaccine signature durability. RhCMV with pp71 deletion has an inhibited ability to undergo cell-to-cell spread, making it a possible safer option to be applied as a future vaccine. Our observations suggest that this spread-deficient phenotype impacts vaccine signature durability. We also note that we observed an increase in polymorphonuclear leukocytes (PMNs) in the attenuated vaccine group compared to the non-attenuated vector group, linking this difference to vaccine attenuation and possibly differences in vector spread in tissue. This attenuation could likely drive a differential inflammatory response that recruits increased PMNs thus displayed as increased circulating neutrophils as they transit from bone marrow to blood to tissue. However, whether maintaining these vaccine-induced cellular and gene expression changes for three years will underlie a differential protection outcome against *Mtb* disease remains unknown until challenge studies with pathological analyses are conducted and completed. A previous RhCMV/SIV study showed that the attenuated pp71-deleted 68-1 RhCMV/SIV vaccine had the same efficacy to protect against SIV infection as the non-attenuated vaccine following virus challenge at approximately three years after last vaccination (6). Ongoing *Mtb* challenge studies of vaccinated cohorts will ascertain vaccine efficacy to protect against *Mtb* disease.

Of particular interest in vaccine response signatures is the presence of the IL-15 response, as previously identified protection signature to RhCMV/SIV vaccination included a robust IL-15 response program that was induced rapidly and persisted in RMs protected from SIV challenge (4, 5, 16, 17). Here we provide the first comparison of RhCMV/TB with RhCMV/SIV vaccine whole blood signatures, revealing that more than 70% of the RhCMV/SIV protection signature genes were also significantly DE in the RhCMV/TB vaccine signatures. These analyses demonstrate that 68-1 RhCMV/SIV and 68-1 RhCMV/TB vector vaccines induce a conserved IL-15 signaling response signature across studies. During the first two years of vaccine phase (for time points D3 through D757) in the current study, our analyses revealed that both the attenuated and non-attenuated 68-1 RhCMV/TB vaccines elicited rapid and stable transcriptional responses after prime administration. This vaccine-induced transcriptional whole blood signature gradually waned during the third year of vaccine phase in RMs administered the attenuated vaccine vector, while the signature induced by the non-attenuated vaccine vector was maintained. Thus, vector persistence with differential cell-to-cell spread may alter the durability of RhCMV whole blood vaccine signatures. An important consideration for the current study is that our observations are limited to whole blood, and the RhCMV vaccine vector induces tissue responses that link with blood cell signatures and effector function to mediate vaccine efficacy. A limitation of our study is that we did not examine the tissue response to vaccination. Importantly, the whole blood vaccine response signature featured the induction of genes involved in cell motility and trafficking, implicating a process in which immune cells are being programmed to migrate into tissues. Thus, tissue responses can occur as an additional component of RhCMV/TB vaccine immune programming that might also impart vaccine efficacy. Our ongoing *Mtb* challenge studies will directly address the tissue response to vaccination to link whole blood and tissue response signature with vaccine efficacy for protection against *Mtb* infection and disease.

IL-15, expressed primarily in myeloid cells (dendritic cells, monocytes, and macrophages), plays an important role in CD8^+^ T cell and NK cell activation (18–22). Previous studies have demonstrated the importance of induction of the unconventionally MHC-E restricted CD8^+^ T cell responses by 68-1 RhCMV vector vaccines for efficacy against SIV (4–6, 17), whereas protection mediated by RhCMV/TB does not specifically require MHC-E restriction (7). We propose that the IL-15 signature is an important component of overall immune programming to promote myeloid cell activation, and NK and CD8^+^ T cell function induced by RhCMV vaccine vectors. Indeed, cell deconvolution analysis showed a vaccination-induced acute increase of NK and CD8^+^ T cells in our vaccine cohorts. We observed a decrease in percentages of these cell types specifically in the attenuated group at later time points (post second boost) concomitant with reduction of the IL-15 signature. Furthermore, the CBC results revealed a similar pattern for the lymphocyte percentage, which supported our transcriptional cell abundance analyses, indicating that maintenance of the IL-15 signature links with vaccine vector-induced NK and T cell abundance in whole blood and could be a determinant of vaccine durability and efficacy.

In conclusion this work shows that the RhCMV/TB vectors elicit a whole blood transcriptional signature with different timing dynamics. Additionally, we link RhCMV vectors with a conserved IL-15 response signature. Future exploration with challenge outcome data and validation will be necessary to examine whether the 68-1 RhCMV/Δpp71-TB-6Ag and other attenuated vaccines protect against TB disease with a strong and stable long-term signature. It is noteworthy that many studies have suggested to use IL-15 as an adjuvant for TB vaccines (4, 23–25) and thus it will be interesting to further investigate the use of 68-1 RhCMV/Δpp71-TB-6Ag vaccine along with IL-15 as an adjuvant to enhance both the protection signature and durability of the vaccine, making it a possible promising vaccine against pulmonary TB disease.

## 4 Material and methods

### 4.1 Rhesus macaques

A total of 24 purpose-bred, pedigreed, RMs (*Macaca mulatta*) of Indian genetic background were included in this non-human primate (NHP) study. Four RMs of 68-1 RhCMV/Δpp71-TB-6Ag group were not included for the further transcriptional analyses due to lack of second boost data. Among these 20 study animals (ten were from the ONPRC colony and the other ten were from the Puerto Rico (PR) colony), 14 were males and six were females, with age ranging from 4 to 7 years old. At assignment, all study RMs were free of *Cercopithecine herpesvirus 1*, D-type simian retrovirus, simian T-lymphotropic virus type 1, simian immunodeficiency virus, and *Mycobacterium tuberculosis*, however, all were naturally RhCMV-infected. All study RMs were housed in Animal Biosafety level (ABSL)-2 rooms during vaccine phase with autonomously controlled temperature, humidity and lighting. Study RMs were both single- and pair-cage housed. Animals were only paired with one another during the vaccine phase if they belonged to the same vaccination group. Regardless of their pairing, all animals had visual, auditory and olfactory contact with other animals. Single cage-housed RMs received an enhanced enrichment plan that was designed and overseen by RM behavior specialists. RMs received commercially prepared primate chow twice daily and received supplemental fresh fruit or vegetables daily. Fresh, potable water was provided via automatic water systems. Physical exams, including body weight and complete blood counts, were performed at scheduled protocol time points. RMs were sedated with ketamine HCl or Telazol for procedures, including subcutaneous vaccine administration and venipuncture.

### 4.2 RhCMV vectors and vaccination

A total of 20 RMs that were included in this transcriptional study were assigned to each of two vaccine groups and vaccinated three times, receiving a prime, a week 14 homologous boost, and followed by a second boost 92 weeks after the first boost. The two vaccines used in this study were 68-1 RhCMV/TB-6Ag vector (n=12) expressing a single six-antigen *Mtb* polyprotein (Ag85A, ESAT-6, Rv3407, Rv2626, Rpf A, and Rpf D) and an analogous RhCMV/TB-6Ag vector (68-1 RhCMV/Δpp71-TB-6Ag; n=8) lacking tegument protein pp71 encoded by RhCMV Rh110 gene (6, 7). The spread-deficient RhCMV/TB vector (ΔRh110) is unable to disseminate from the injection site to distant sites *in vivo*, but it remains immunogenic for T cell responses (26). The 68-1 RhCMV/TB vector was constructed by bacterial artificial chromosome (BAC) recombineering, and the vector preparations and characterization have been previously described (3, 6, 27–29). In this study, RMs were vaccinated subcutaneously at a dose of 5×10^6^ plaque-forming units (PFU) of each of the assigned 68-1 RhCMV/TB vectors.

### 4.3 Sample collection, library prep, and RNA sequencing

Longitudinal whole blood samples were collected into PAXgene RNA tubes (PreAnalytiX) following the manufacturer’s instructions. RNA was isolated using RNAdvance Blood Kit (Beckman) on a Biomek i7 Automated Workstation (Beckman) following the validated protocol provided with the kit that included an on-column DNase treatment. Total RNA was quantified using Qubit™ RNA Assay kit, and RNA integrity assessed using the Agilent 4200 TapeStation instrumentation. mRNA-seq libraries were constructed using the KAPA HyperPrep Kit with RiboErase (HMR) Globin + mRNA Capture Kit (Roche Diagnostics) following the manufacturer’s recommended protocol that was adapted for the Biomek i7. Samples were randomized across the project, including internal plate controls to protect against batch effects. Qubit™ DNA Assay Kit was used to determine library DNA concentration and Agilent TapeStation assay was used to determine library size distribution and quality. Libraries were sequenced on an Illumina NovaSeq X sequencer at Northwest Genomics Center (University of Washington) using an Illumina NovaSeq S2 v2 200 cycle kit following the manufacturer’s protocol for sample handling and loading. Sequencing run metrics were visualized for quality assurance followed by demultiplexing using bcl2fastq. A total of 640 fastq files were generated for downstream transcriptomic analyses, and the quality were assessed using FastQC.

### 4.4 Sequence data pre-processing and preparation for analysis of differential expression

Raw sequence reads were processed to digitally remove residual adapters and low-quality bases by using Trim Galore v0.6.4 (powered by Cutadapt v3.7 (30);), along with quality check using FastQC v0.11.2 (31). Trimmed reads were then filtered to remove globin and ribosomal sequences using Bowtie2 v2.3.4 (32). Paired reads were aligned to the Mmul_10 *Macaca mulatta* genome (Ensembl v109 (33);) and gene expression was quantified with STAR v2.7.10b (34) using the –quantMode GeneCounts flag to generate raw gene counts. After removing lowly expressed genes with mean gene counts below 4, trimmed mean of M-values (TMM (35);) normalization was performed using the calcNormFactors function in edgeR (36) to calculate the scaling factor for the adjusted library sizes. Gene counts were then transformed into log_2_-counts per million (log-CPM) values by limma package (37) with the voom function (38) for subsequent analyses.

### 4.5 Principal component analysis

Principal component analysis (PCA) was performed on the transformed normalized count matrix for an initial inspection of the whole transcriptome. This allows visualization of samples to determine sources of variation across samples within a dataset to include as a covariate in the final model. Eigenvalues were computed using the built-in R function prcomp (39–41) with all expressed genes, and the top two eigenvectors represent the two dimensions on the PCA plot. Biplots with the top two principal components, PC1 and PC2, were generated using data points from all 320 samples (including 20 RMs, with 16 time points each) with the label of vaccine group, sex, and time point.

### 4.6 Deconvolution analysis

To further impute the gene expression profiles with an estimation of cell type proportions, we input normalized gene expression values for all samples into CIBERSORTx (9), a machine learning tool, to predict cell abundance levels. We represented similar cell types together by summing predicted cell percentages as the following: B cells naive and B cells memory as B cells, T cells CD4 naive, T cells CD4 memory resting and T cells CD4 memory activated as CD4 cells, NK cells resting and NK cells activated as NK cells, and dendritic cells resting and dendritic cells activated as dendritic cells.

### 4.7 Differential gene expression analysis and visualization

Differential gene expression analysis was performed for each time point using D0 as the common baseline comparator of each vaccine group. To determine the significant differentially expressed (DE) genes in the RhCMV/TB vector-vaccinated cohort, normalized counts were utilized for linear modeling across contrasts for each gene using the limma package in R and Bioconductor (42). Significant DE genes were identified with a false discovery rate (FDR; BH procedure)-adjusted *p*-value less than 0.05 and an absolute Log_2_ fold change (LFC) value greater than 0.58. These genes were then organized by Pearson correlation method for the calculation of correlation-based distance metrics, followed by grouping into co-expression modules using the Ward.D2 hierarchical clustering method. Heatmaps were generated for the visualization of the LFC values of clustered DE genes using the WGCNA (43, 44), heatmap.2 (codes on GitHub) with gplots, and edgeR (36). Further graphics were made using R packages, including ggplot2 (45) for barplots to display the numbers of DE genes at each time point, and eulerr (46, 47) for the proportional Venn diagram.

### 4.8 Over representation analysis

Functional enrichment analysis was performed for each module of DE genes using over representation analysis (ORA) with the enrichGO function in the clusterProfiler package (48) to determine whether genes that were pre-defined to be associated with certain annotations are statistically more prevalent in our gene sets of interest than would be expected by chance. The biological process subontology of Gene Ontology (GO: BP) annotations used in this study were retrieved from org.Hs.eg.db (49). After converting the RM Ensembl gene IDs to Human Genome Organisation Gene Nomenclature Committee (HGNC) gene symbols, significantly over-represented genes were identified using a threshold of FDR-adjusted *p*-value less than 0.05. Network plots were generated using Cytoscape (50) for the visualization of representative biological processes and the significantly enriched DE genes.

### 4.9 Upstream regulator analysis

Upstream regulator analysis was performed using Ingenuity Pathway Analysis (IPA; QIAGEN Inc (51).;). The LFC values and FDR-adjusted *p*-values of all the significant DE genes were input for the analyses, and the information of the top identified upstream transcriptional regulators and cytokines that were common across time points in both vaccine groups were collected for making dot plots using ggplot2 in R. Networks were generated through Cytoscape from IPA results. We extracted the significant upstream regulators and their target genes, generate a network file for each time point and mapped the LFCs for each vaccine vector to the corresponding genes using the Omics Visualizer app within Cytoscape.

## Data Availability

The raw data is available on GEO GSE273911. The R codes applied to these analyses can be accessed at https://github.com/galelab/Sung_RhCMV_TB_IL15_Attenuation.

## Supplementary material

Supplementary Table 1 Animal information (vaccine, sex, and age).

Supplementary Table 2 Global DE genes information (LFC, adjusted *p*-value, and cluster numbers).

Supplementary Table 3 IL-15 responsive protection signature and the overlapping genes.

Supplementary Table 4 Global enrichment information with associated genes.

Supplementary Table 5 Top upstream regulators information (z-score and *p*-value).

Supplementary Table 6 Four interleukin genes information (LFC and adjusted *p*-value).

Supplementary Table 7 Conserved DE genes information (LFC, adjusted *p*-value, and cluster numbers).

Supplementary Table 8 Conserved enrichment information with associated genes.
